# ‘Two Pains Together’: Patient Perspectives on Psychological Aspects of Chronic Pain while Living with HIV

**DOI:** 10.1371/journal.pone.0111765

**Published:** 2014-11-03

**Authors:** Jessica S. Merlin, Melonie Walcott, Christine Ritchie, Ivan Herbey, Stefan G. Kertesz, Eric Chamot, Michael Saag, Janet M. Turan

**Affiliations:** 1 Division of Infectious Diseases, Department of Medicine, University of Alabama at Birmingham, Birmingham, Alabama, United States of America; 2 Division of Gerontology, Geriatrics, and Palliative Care, University of Alabama at Birmingham, Birmingham, Alabama, United States of America; 3 Division of Geriatrics, Department of Medicine, University of California at San Francisco, San Francisco, California, United States of America; 4 Jewish Home of San Francisco Center for Research on Aging, San Francisco, California, United States of America; 5 Birmingham VA Medical Center, Birmingham, Alabama, United States of America; 6 Division of Preventive Medicine, Department of Medicine, University of Alabama at Birmingham, Birmingham, Alabama, United States of America; 7 School of Public Health, University of Alabama at Birmingham, Birmingham, Alabama, United States of America; University of Texas Medical Branch, United States of America

## Abstract

**Objective:**

Chronic pain is common in HIV-infected individuals. Understanding HIV-infected patients’ chronic pain experience not just from a biological, but also from a psychological perspective, is a critical first step toward improving care for this population. Our objective was to explore HIV-infected patients’ perspectives on psychological aspects of chronic pain using in-depth qualitative interviews.

**Methods:**

Investigators engaged in an iterative process of independent and group coding until theme saturation was reached.

**Results:**

Of the 25 patients with chronic pain interviewed, 20 were male, 15 were younger than age 50, and 15 were African-American. Key themes that emerged included the close relationship between mood and pain; mood and pain in the context of living with HIV; use of alcohol/drugs to self-medicate for pain; and the challenge of receiving prescription pain medications while dealing with substance use disorders.

**Conclusions:**

The results suggest that psychological approaches to chronic pain treatment may be well received by HIV-infected patients.

## Introduction

Chronic pain is defined as pain of at least 3 months’ duration, not associated with ongoing tissue injury. [1] Chronic pain in HIV-infected patients is a common, disabling condition. It occurs in between 39–85% of HIV-infected patients,[2]–[4] and HIV-infected patients with chronic pain have up to 10 times greater odds of functional impairment. [5] Chronic pain often occurs in the context of medical, psychiatric, and addiction comorbidities, which add an additional layer of complexity for both the patient and provider.[6]–[8].

The clinical approach to chronic pain has historically drawn from a biomedical model. [9] For example, despite limited evidence of efficacy, chronic opioid therapy is commonly prescribed in both the general population and in HIV-infected patients. [10] [11] Psychological approaches such as cognitive behavioral therapy [12] and pain self-management [13], [14] have been used in the general population with promising results in randomized controlled trials. [15] However, such interventions have not seen widespread implementation and dissemination and are often inadequately reimbursed. Furthermore, psychological interventions have not been adapted to HIV-infected patients who are likely to have a unique chronic pain experience, due to unique HIV-related biological factors, complex social challenges, and high degrees of medical, psychiatric, and addiction comorbidity. [16].

The Biopsychosocial (BPS) Framework is an explanatory conceptual framework based on systems theory that has been applied to medical and psychiatric illnesses, [17] including chronic pain. [18] Understanding HIV-infected patients’ chronic pain experience not just from a biological, but also from a psychological and social perspective, is a critical first step toward improving their care. Therefore, we recently adapted and tailored this framework to HIV-infected patients with chronic pain by identifying factors common to both conditions. [16] Our goal in the current manuscript is to explore patients’ perspectives on the psychological aspects of pain, using the psychological portion of the BPS framework as a guide.

## Methods

### Study Design

We conducted qualitative interviews, and used individual interviews as the topics covered were of a sensitive nature, and we sought to obtain in-depth information regarding patient perspectives on the chronic pain experience. Our study methods have been described in detail previously. [19].

### Research Context

This study took place at a comprehensive HIV clinic, which includes HIV primary care and psychiatric and addiction services, in an urban center in the Southern US. Most clinic patients participate in the Center for AIDS Research Network of Integrated Clinical Systems (CNICS) study, which involves completion of Patient Reported Outcome (PRO) measures every 6 months on touch-screen computers.

### Inclusion criteria and sampling

Inclusion criteria were a) age ≥19 (Alabama’s legal age of majority), b) participation in CNICS, and c) at least mild chronic pain based on a Brief Chronic Pain Screening tool (BCPS). [19] Participant selection was also designed to ensure representation of “patients who are likely to be the target of future chronic pain interventions, and who are vulnerable to worse pain-related outcomes.”[19]–[21] Therefore, we used PROs to purposively sample individuals with *and* without various symptoms of a major mood disorder, defined as depressive symptoms (PHQ-9≥10) or anxiety symptoms/panic (PHQ-Anxiety module), [22] and illicit substance use (ASSIST, excluding marijuana) (23). Participants provided informed consent and were reimbursed $20.

### Interview Design and Data Collection/Analysis

The qualitative interview guide was based on the BPS Framework for Chronic Pain in HIV (Table 1) and modified emergently during the study. Hour-long interviews were conducted by an experienced qualitative interviewer (MW), audio-recorded, transcribed verbatim, and coded using NVivo 10.0 while interviews were still ongoing. Transcripts were initially coded by IH, who developed the codebook while remaining blinded to the interview guide. Subsequently, three investigators (JSM, MW, IH) engaged in an iterative process of independent and group coding. Interviews were conducted until theme saturation was reached. Results presented are limited to psychological themes, and to participants who reported at least mild chronic pain as defined above.

**Table 1:** In-Depth Interview Guide Psychological Probes.

Psychological distress	Tell me more about your mood. Does pain affect your mood? (Probe: do you think that the people close to you think that pain affects your mood, or detect changes in your mood related to pain?)
Substance use	How do you think using drugs or alcohol is related to pain? What effect do you think drug or alcohol use has on your pain?
Environmental stressors	Is pain affected by other things that cause stress in your life? (Probe: your job, your family, money, housing situation, access to food, etc? If so, how?)
Anger	In our everyday lives, there are things that make us angry. Do these things affect your pain? (Probe: if so, how?)
Fear/Traumatic life events	Have you ever been in any situation that was extremely frightening or horrifying, or one in which you felt extremely helpless? (Probe: If yes, what was it? Your chronic pain started X years ago-was that before or after this event?)

### Ethics Statement

This protocol was approved by the University of Alabama at Birmingham’s Institutional Review Board, which specifically approved this study. All participants provided written informed consent.

## Results

Of the 25 participants, 5 were female, 10 were age 50 or greater, 15 were African-American, the median CD4+ T-cell count was 571 cells/mm3, and 20 had an undetectable viral load (Table 2). The majority (16) had a mood disorder, and nearly half (12) reported actively using illicit substances. Six reported mild pain, 4 reported moderate pain, and 14 reported severe pain (1 did not report pain severity).

**Table 2:** Characteristics of Participants with Chronic Pain, as identified by the Brief Chronic Pain Screening Tool (BCPS) Results (N = 25).

	Mild-moderate-very severe pain for at least 3 months (N = 25)*
Female	5
African-American	15
Age ≥50	10
CD4+ T-cell count (median, IQR)	587 (421–792) cells/mL
VL <200 copies/mL	14
Pain**	25
Mood disorder†	16
Substance use††	12

*BCPS results typically dichotomized as mild-moderate-severe pain for at least 3 months ( = chronic pain), and none or any pain less than three months ( = not chronic pain).

**Based on the EuroQOL pain measure indicating mild, moderate or severe pain within the 6 months prior to enrollment.

† Based on a PHQ-9≥10 or a PHQ-Anxiety module consistent with anxiety symptoms/panic within the 6 months prior to enrollment.

†† Based on an ASSIST reporting use of opiates, heroin, cocaine, or amphetamines within the 6 months prior to enrollment.

Major psychological themes that emerged were mood and relationships between alcohol/illicit substance use and pain.

### MOOD (Depression and Anxiety)

Themes that emerged in this category were: a) the close relationship between mood and pain, and b) mood and pain in the context of living with HIV.

#### a) Close relationship between mood and pain

Many participants described a strong connection between mood – mostly depression, but also anxiety – and pain. One participant’s comment clearly articulates the challenging nature of the coexistence of mood disturbance and pain:

*I mean because I’m already feeling down, and then my pain makes me ache. And then I’m down and I’m aching. That’s two pains together. That’s not good.* 49-year-old African American male

The three main sub-themes that emerged regarding the close relationship between mood and pain were: 1) bidrectionality, 2) the relationship between physical and emotional pain, 3) coping with mood disturbance and pain.

##### 1. Bidirectionality

The connection between mood and pain was often explained as bidirectional. Specifically, participants explained that having pain can exacerbate pre-existing mood disorders, and that difficulties with mood can also make pain worse. For example:

*I really have to work on my depression, and the depression gets worse when the pain gets worse. … I know that for a fact. There’s no two ways about it. The pain gets worse, so does the depression…. And it’s hard to handle both of ‘em at one time. …. I’ve been suffering with [depression] about as long as I have the pain…. And it – as the day goes on, the pain gets worse. The depression gets worse. …. I tell you living in pain everyday, it'll – it depressed the hell out of ya.* 56-year-old white male

##### 2. Relationship between physical and emotional pain

Participants readily described the concept of not only physical, but also emotional pain. Their comments indicate that physical and emotional pain are two very closely related constructs. Some participants described that at times, physical and emotional pain are indistinguishable:

*Interviewer: What is the relationship between your depression and your pain? …*

*Participant: Both of them are the same to me. … I be depressed all the time, every day. The pain is the same way. I be in pain every day, all day.* 46-year-old African-American female

*Participant: When you consider peoples' pain, a lot of it is emotional…. If you're happy your bones are happy…. If you’re sad your bones are sad.* 49-year-old African-American male

Still others explained that emotional distress actually creates a painful sensation:

*Participant: Uhm, and it’s just you know, with depression comes just, it’s like almost a weight, uhm, uhm, or pressure that can translate to, to physical pain at times…when it gets bad … I don’t know if it, if it’s something that I would necessarily always refer to as, as a physical pain more than an emotional pain. But at times, sometimes that, that line between the two becomes a little blurred. … And it, uhm, it’s kind of hard to tell what exactly hurts.* 31-year-old white male

However, a few participants drew a sharp distinction between physical and emotional pain:

*Participant: The depression really is painful. Does that make sense to you? … You know, there is a different kind of pain than your leg hurting, you know?* 37-year-old white male

##### 3. Mechanisms for relationship between mood and pain

Participants offered some potential mechanisms to explain the relationship between pain and mood. These related to effects on physical and social functioning. In describing this relationship some participants noted that worse mood contributes to reluctance to participate in physical or social activities, leading to impaired physical and social function. Others noted that depression independently leads to impaired function, and decreased function further worsens pain.

Another participant explained the relationship between his anxiety and pain, and the mediating role of depression:

*Anxiety affects my pain by keeping me from doing things like driving and things like that, that I really shouldn’t be afraid of doing…. That makes me depressed, so that makes me want to just sit and muddle in my pain.* 50-year-old white male

On the flip side, a few participants acknowledged that being in a good mood helped them to cope with their pain better:

*If I’m in a good mood uh I can kinda deal with [pain] more…. If I’m in a bad mood it’s just like nothing else really matters other than, you know, tryin’ to get comfort.* 48-year-old African-American male

#### b) Mood and Pain in the Context of Living with HIV

The majority of participants appeared to conceptualize their HIV and their chronic pain as two distinct health conditions and did not attribute their pain to their HIV, and did not think that their HIV caused pain. However, some participants reported that having HIV is depressing, having pain is depressing, and the combined effect of having both at the same time can be difficult to manage:

*I am hurting all the time and hurting all the time when you have HIV and stuff like that you want to live a normal life but you know you cannot live a normal life. And sometimes you are out there and you forget about it and then reality kicks in and you have to say whoa, pull yourself back.* 47-year-old African-American male

*Well, just I've got enough problems dealing with HIV, and trying to get myself clean, and then you throw in the pain on top of that, well, that's just something I don't need, right now. And it just I mean, you can only take so many hard licks from one day, before you just gotta give up. And, if you got that damn pain sitting there, just gnawing, it's like somebody's gnawing on a bone, and you got that sitting there.* 56-year-old white male

### Relationships between Alcohol/Illicit Substance Use and Pain

Another category that emerged initially and was then specifically probed was the relationship between alcohol/illicit substance abuse and pain. In this category, themes that emerged were: a) the use of alcohol/drugs to self-medicate for pain, and b) the challenge of receiving prescription pain medications while dealing with current or past history of substance use disorders.

### a) Use of alcohol/drugs to self-medicate for pain

A minority of participants reported that pain actually led them to use alcohol or drugs, in an attempt to alleviate their suffering. One participant described how in his case alcohol worked to help him manage his pain:

*I would just drink [alcohol] it until my mind – it takes your mind differently. After you’ve had a few you get a good buzz going on and you don’t think about the pain no more.* 34-year-old African-American male

Another participant explained how pain actually fueled the development of his addiction to illicit drugs:

*Well, [pain and drug use] go hand in hand. Because when I hurt I wanna stop hurting so… and I know that… I know what will help me stop hurting…But and that’s the problem now is I use it when I’m not hurting…. It did go hand in hand for me but now it just became a, a problem or addiction.”* 45-year-old African-American male

Most participants who discussed the role of illicit substances felt that self-medication in this way is only a temporary fix. After the drug has worn off, the person is left with their original pain:

*You know, you know, drugs doesn’t help because uh if you try to alleviate the pain with – with drinkin’ alcohol ‘cause you gonna just – it’s gonna be a hurtin’ drunk.* 52-year-old African-American male with severe chronic pain

*For the few minutes that I’m using drugs, I don’t hurt…. And I might, uh, it might last, the pain might be gone for thirty minutes, hour…. But that’s only because cocaine done numb the feeling, I guess…But it, it always comes back which caused me to keep going get more cocaine* 45-year-old African-American male

One participant felt that after getting off drugs his pain was even worse:

*Uh actually … when I was doin’ the drugs [crack cocaine] I – I didn’t notice the pain. But now that I’m not I do feel – feel it a little more intensely.* 48-year-old African American male

#### b) Prescription pain medication and substance use

Participants reported that clinicians are reluctant to prescribe opioids for individuals with substance use, often discovered on a urine drug screen. In some cases, this caused frustration, as the patient felt they really needed medications to deal with the pain.

Several participants reported their own fear of receiving prescription pain medications due to a personal history of substance use. They feared that the pain medications would start them back on the road to addiction.

*Those pills, that methadone, that’s a substance to me. To me that’s a substance. I don’t want that…. When they gave me that first, I told that Dr. X, because he put me on that first to see how I would do. I told him “No, I don’t want that.” I don’t want it.* 49-year-old African-American male

*I don’t wanna get back on drugs again…. I don’t want ‘em treatin’ me with narcotics…. Because I been on ‘em before and I don’t want back on ‘em….*

*Interviewer: You think the doctors would treat you with narcotics again?*

*Participant: If I wanted ‘em to probably. … I’m not gonna do it…Back when I was gettin’ my teeth pulled down here he was givin’ me Lortabs for pain. I didn’t take ‘em. I didn’t take ‘em. I just took aspirins ‘cause I didn’t wanna get strung out again…I cut my hand not long ago and had to go get stitches in it out in the yard. And she gave me a bunch a Lortabs for pain, that doctor did. I didn’t – I didn’t take ‘em. I don’t even remember if I filled the script. I mighta filled the script once or twice but a lot of ‘em I didn’t fill just in case it got to hurtin’ real bad. But I didn’t take ‘em.* 51-year-old white male

On the flip side, one participant understood the clinicians’ logic, but nevertheless wished they would prescribe:

*They won’t put me on narcotics, ‘cause I’m – I was addicted to crack cocaine for a while…So, they an – they’re not real enthusiastic about giving me the dem – uh the uh, hydrocodone…So, just have to suffer. …Well, [SIGH] I – you know, I can see the reason, because I am an addict, and I always will be. But I was an addict to crack cocaine…. I just don't really see why they can't give me the narcotics now, because that's what gets me out of the pain, and I wouldn't want but one a day, because I can – I can take it up to twelve to one o’clock, but after twelve to one o'clock, I could take that, and I would be pain free the rest of the night. But because I'm an addict, you know, and I can see where they're coming from, you know, because but uh, and I've got several cousins, and uh, they've all been addicted to pain killers, and I wouldn't wanna get in their shape. Their livers are bad. Their uh, they're always chasing the dope, and I know how that comes in to being, cause I chase crack before, but uh, it's either stay in pain, or take the narcotics.* 56-year-old white male

Another participant also drew a direct link between the use of opioids for pain and the development of addiction to illicit substances:

*Lortabs gets you started…Then once you can’t get them anymore you go out there and find that heroin or that Dilaudid… Because uh the more drugs they give ya to treat your pain the worse off you get…It’s just a spiral down…Before long it’s all you want, that’s all you want. That’s all you gotta have. Don’t care about nothin’ else. Don’t wanna do nothin’ else…You just want that next shot.* 51-year-old white male

## Discussion

This is the first study to focus on psychological aspects of chronic pain among HIV-infected individuals. Our results highlight the importance of the relationship between chronic pain, mood, and substance use in a sample of HIV-infected individuals enriched with individuals with mood disorders and substance use.

This study is consistent with previously published evidence that mood disorders, particularly depression, are common and complex chronic diseases in individuals with HIV. [23] The high level of comorbidity between depression and chronic pain in the general population has also been well-established. [24] Additionally, there are studies that suggest that patients with severe depression and chronic musculoskeletal pain, [25] fibromyalgia, [20] and low back pain [26] have worse functional outcomes, and that depression severity mediates the role of post-traumatic stress disorder on pain interference. [27] The close, often bidirectional relationship between mood and pain that patients described has also been observed quantitatively. [14] Furthermore, our patients expressed a nuanced understanding and candidness about this relationship, and about the concepts of both physical and emotional pain.

Historically, providers have addressed pain mainly from a biomedical perspective, [9] focusing on the importance of gathering objective evidence of biological causal factors. [28] In our experience, providers assume that patients are equally wedded to this biomedical model, and are not as open to acknowledging psychosocial influences. Patients’ interest in operating within the biomedical model may relate to how providers use diagnostic testing in patients with chronic pain. Patients often observe providers who use positive diagnostic test results to confirm the existence of the pain, and the lack of a positive diagnostic test to cast doubt on the pain’s existence. [29] Prior studies have suggested that while providers focus on convincing patients with negative test results that there is no biological basis for their pain, patients insist that there is a biological basis in order to gain credibility. [30].

Patients in our study also clearly articulated the relationship between pain and substance use. They acknowledged the practice of self-medicating using alcohol and illicit substances, the ineffectiveness of this approach, and the risk of addiction. Patients’ willingness to address these issues was recently echoed in another study of physician-patient communication regarding chronic opioid therapy. [31] Despite patients’ ability to discuss topics related to pain and addiction, HIV primary care providers often feel unprepared to address opioid misuse and addiction in patients with chronic pain. [32].

Our study suggests that patients make a direct connection between mood, alcohol/substance use, and pain, and seem to understand the way in which these factors work together to impact their health. Providers may feel that such conversations may be interpreted as telling patients that their pain is “all in their head” [33]. While statements such as that should certainly be avoided, an open-ended, non-judgmental approach to discussing the psychosocial aspects of pain, similar to that used in this study, could facilitate provider-patient communication about this difficult topic. Further research into effective communication strategies about chronic pain between patients and providers is needed. [34].

Despite the importance of psychological aspects of pain, the most readily available pain treatments are biomedical, and do not address mood or substance use. For example, the most commonly prescribed medications for pain in the US are opioids, which have increased in use dramatically over the past 10 years. [10] Opioids are more commonly prescribed for HIV-infected patients with chronic pain than HIV-uninfected patients, [11] and HIV-infected individuals are more likely to receive high dose opioids. [35] Despite their frequency and intensity of use, a recent Cochrane review concluded that among participants who continued the opioid, results were insufficient to draw conclusions regarding function, and there was only weak evidence to substantiate improvement in pain. [36] Likewise, HIV-infected individuals with chronic pain on chronic opioid therapy have worse pain than those not on chronic opioid therapy. [37] In addition to these major efficacy concerns, opioids carry serious risks, including misuse and overdose.[38]–[42] This is especially concerning in patients with a history of substance use – a common comorbidity in HIV-infected patients. [43].

Our study suggests that HIV-infected patients understand the limited efficacy of both licit and illicit substances in the management of pain, and would welcome a behavioral approach addressing the psychological aspects of pain highlighted here. Evidence-based behavioral interventions are among the most effective non-pharmacologic chronic pain treatments investigated in the general population. [24] While most participants in our study did not perceive their pain to be caused by their HIV, they did underscore the importance of chronic pain in the context of living with HIV. Other studies have shown that the prevalence and complexity of depression is higher among HIV-infected patients than in the general population. [23], [44] Based on our findings, depression may actually play a key role in the relationship between HIV and chronic pain. This supports existing knowledge and practice and affirms that treating depression and incorporating psychological approaches to pain management is critical in HIV-infected patients. Therefore, given the importance of the context of HIV, chronic pain behavioral interventions specifically tailored to HIV-infected patients, particularly ones that incorporate treatment of depression and substance abuse, should be developed.
